# Comparative genomics of *Roseobacter* clade bacteria isolated from the accessory nidamental gland of *Euprymna scolopes*

**DOI:** 10.3389/fmicb.2015.00123

**Published:** 2015-02-23

**Authors:** Andrew J. Collins, Matthew S. Fullmer, Johann P. Gogarten, Spencer V. Nyholm

**Affiliations:** ^1^Molecular and Cell Biology, University of ConnecticutStorrs, CT, USA; ^2^Microbiology, The Forsyth InstituteCambridge, MA USA; ^3^Institute for Systems Genomics, University of ConnecticutStorrs, CT, USA

**Keywords:** symbiosis, *Euprymna scolopes*, *Roseobacter* clade, genomics, Cephalopoda, *Alphaproteobacteria*

## Abstract

The accessory nidamental gland (ANG) of the female Hawaiian bobtail squid, *Euprymna scolopes*, houses a consortium of bacteria including members of the *Flavobacteriales*, *Rhizobiales*, and *Verrucomicrobia* but is dominated by members of the *Roseobacter* clade (Rhodobacterales) within the *Alphaproteobacteria*. These bacteria are deposited into the jelly coat of the squid’s eggs, however, the function of the ANG and its bacterial symbionts has yet to be elucidated. In order to gain insight into this consortium and its potential role in host reproduction, we cultured 12 Rhodobacterales isolates from ANGs of sexually mature female squid and sequenced their genomes with Illumina sequencing technology. For taxonomic analyses, the ribosomal proteins of 79 genomes representing both roseobacters and non-roseobacters along with a separate MLSA analysis of 33 housekeeping genes from *Roseobacter* organisms placed all 12 isolates from the ANG within two groups of a single *Roseobacter* clade. Average nucelotide identity analysis suggests the ANG isolates represent three genera (*Leisingera*, *Ruegeria*, and *Tateyamaria*) comprised of seven putative species groups. All but one of the isolates contains a predicted Type VI secretion system, which has been shown to be important in secreting signaling and/or effector molecules in host–microbe associations and in bacteria–bacteria interactions. All sequenced genomes also show potential for secondary metabolite production, and are predicted to be involved with the production of acyl homoserine lactones (AHLs) and/or siderophores. An AHL bioassay confirmed AHL production in three tested isolates and from whole ANG homogenates. The dominant symbiont, *Leisingera* sp. ANG1, showed greater viability in iron-limiting conditions compared to other roseobacters, possibly due to higher levels of siderophore production. Future comparisons will try to elucidate novel metabolic pathways of the ANG symbionts to understand their putative role in host development.

## INTRODUCTION

The *Roseobacter* clade is a pervasive and diverse group of marine *Alphaproteobacteria.* This group is estimated to account for 10% of all marine bacteria, with higher percentages in coastal seawater (Wagner-Döbler and Biebl, 2006). These organisms have usually been investigated from an ecological perspective due to their abundance in seawater. The combined metabolic potential of such a large bacterial population may contribute to both sulfur cycling, primarily through metabolism of dimethylsulfoniopropionate (DMSP), and carbon cycling, as roseobacters oxidize a variety of carbon sources to CO_2_ (González et al., 2000).

**FIGURE 1:**
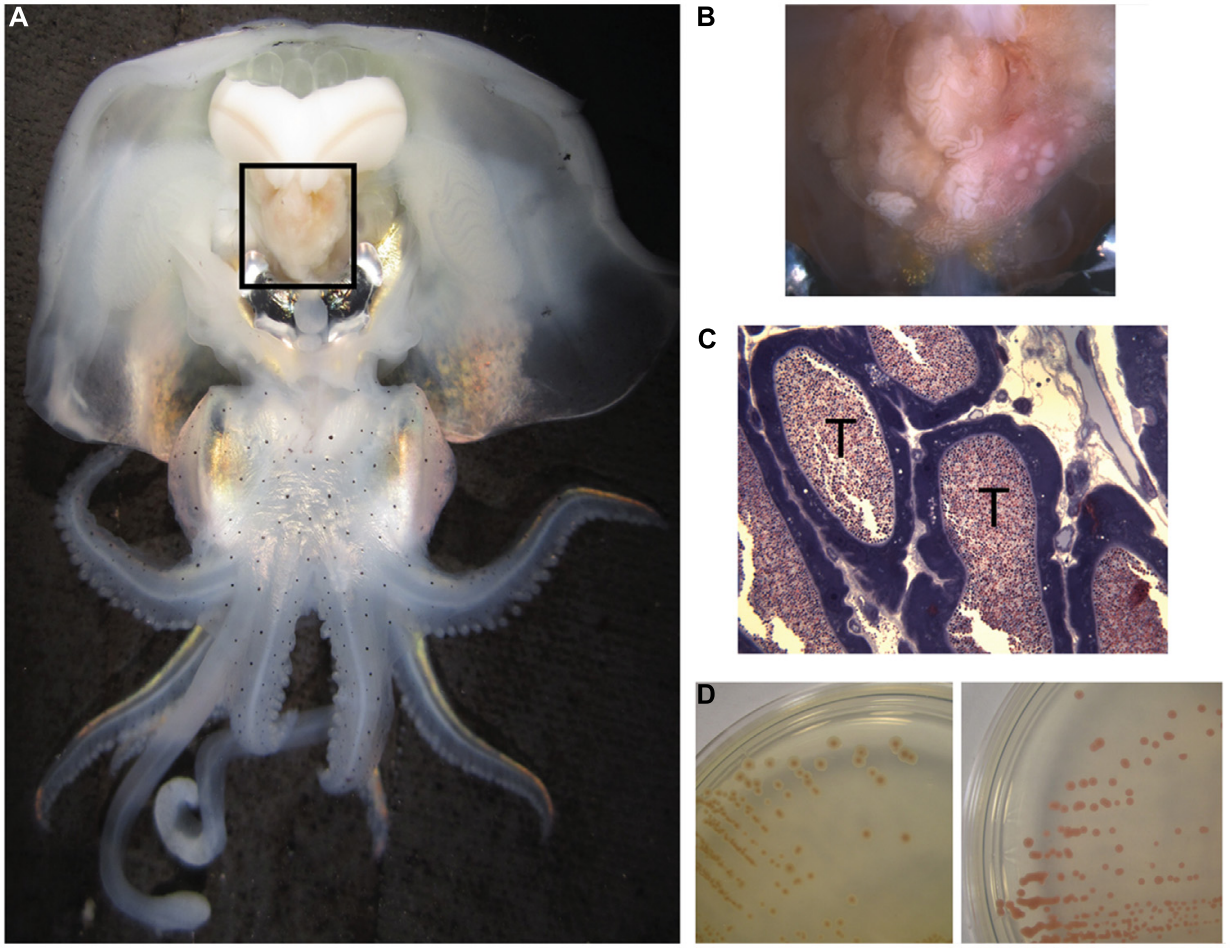
**Anatomy of *Euprymna scolopes*, the accessory nidamental gland (ANG) and associated bacteria. (A)** The ANG (black box) is located on the ventral side of the squid, posterior to the ink sac and light organ. **(B)** The ANG is comprised of many pigmented convoluted epithelium-lined tubules that may appear white, pink, or brown. **(C)** Microscopy of fixed and stained sections of the ANG reveal dense populations of bacteria within tubules (T). **(D)** Bacteria from the ANG that were isolated on complex media also had a variety of pigments, from brown (*Leisingera* sp.ANG-Vp, left) to red (*Tateyamaria* sp. ANG-S1, right).

Many of the characterized *Roseobacter* isolates can be described as free-living, having been isolated from seawater or inert marine surfaces. However, some roseobacters also associate with other organisms, including oysters (Ruiz-Ponte et al., 1998), sponges (Zan et al., 2014), algae (Rao et al., 2007; Case et al., 2011), and cephalopods (Grigioni et al., 2000; Pichon et al., 2005; Collins et al., 2012). Among many squid and cuttlefish, roseobacters have been found associated with the accessory nidamental gland (ANG), part of the female reproductive system and comprised of many epithelium-lined tubules that house dense populations of bacterial symbionts (**Figure 1**, Bloodgood, 1977; Collins et al., 2012). Evidence suggests that these bacteria are embedded in the jelly coat of the squid’s eggs that are then deposited in masses on the ocean floor where they resist fouling and degradation over ~3 weeks of development (Barbieri et al., 2001; Collins et al., 2012).

Studies that have investigated the ANG consortium have found members of the *Roseobacter* clade among many cephalopods, including *Doryteuthis pealeii, Sepia officinalis,* and *Euprymna scolopes* (Grigioni et al., 2000; Barbieri et al., 2001; Pichon et al., 2005; Collins et al., 2012). In the Hawaiian bobtail squid, *E. scolopes,* roseobacters comprise ∼50% of the microbial population according to 16S rDNA surveys, predominantly from the genus *Leisingera* (formerly *Phaeobacter*; Collins et al., 2012). Other members of the consortium include *Flavobacteria* and *Verrucomicrobia* and each of these groups are partitioned such that only one taxon dominates any given tubule (Collins et al., 2012).

*Roseobacter* clade bacteria are known to produce several antimicrobial compounds, including tropodithietic acid (TDA), which has antimicrobial and anti-algal properties (Brinkhoff et al., 2004). Under certain conditions, likely when associated with dying algae, *Phaeobacter inhibens* can also produce anti-algal compounds known as roseobacticides derived from *p*-coumaric acid, a product of lignin degradation (Seyedsayamdost et al., 2011). *Leisingera* sp. Y4I and *Leisingera daeponensis* produce indigoidine, an antimicrobial blue pigment that is synthesized from a unique polyketide/non-ribosomal peptide synthase gene cluster and has been shown to inhibit marine bacteria, including *Vibrio fischeri* (Cude et al., 2012; Dogs et al., 2013).

The function of the ANG and its associated bacterial population remains unknown although protective roles against predation and/or fouling have been suggested (Biggs and Epel, 1991). The distribution of roseobacters among cephalopod ANGs suggests that they have a conserved function in these animals. Furthermore, they must contain traits that allow them to survive in multiple habitats such as seawater, a specialized organ such as the ANG, and within squid egg jelly coats. To shed light on the metabolic capabilities of these bacteria and investigate possible adaptations to living in these different habitats, we examined the genomes of 12 isolates from the ANG of *E. scolopes* and compared them to others from the *Roseobacter* lineage. Here, we describe the genetic content from this select group of roseobacters that exist in conserved symbioses with cephalopods worldwide.

## MATERIALS AND METHODS

### CULTURING BACTERIA FROM THE ANG

Animals were collected in sand shallows on Oahu, Hawaii and maintained in artificial aquaria as previously described Schleicher and Nyholm (2011). To obtain ANGs, five mature females were anesthetized in Instant Ocean with 2% ethanol. Organs were removed and surface sterilized with 70% ethanol before being homogenized in filter-sterilized squid Ringer’s solution (530 mM NaCl, 25 mM MgCl_2_, 10 mM CaCl_2_, 20 mM HEPES, pH = 7.5). Tissue homogenate was serially diluted and plated on either salt water tryptone (SWT) or Reasoner’s 2A medium (R2A) supplemented with a 70:30 mixture of Instant Ocean and distilled water (Reasoner and Geldreich, 1985; Nyholm et al., 2009). Plates were incubated aerobically at 28°C for 2–7 days. For each animal, colonies with different morphology and/or color were isolated for further analysis.

### GENOME SEQUENCING AND ANNOTATION

Genomic DNA was isolated using the MasterPure DNA Extraction kit (Epicentre) from liquid cultures of ANG bacteria grown overnight at 28°C in either SWT or R2A. DNA was quantified using a Qubit fluorescence assay (Invitrogen). Illumina sequencing libraries were created from 1 ng of genomic DNA using the Nextera XT library kit and the libraries were quantified by a HS DNA Bioanalyzer assay (Agilent). Libraries were sequenced on an Illumina MiSeq sequencer using 2 × 250 bp reads. Draft genomes were assembled using the CLC Genomic Workbench (CLC) using default parameters. For *Leisingera* sp. ANG1 (formerly *Phaeobacter gallaeciensis* ANG1), additional sequencing data was added from a previous sequencing effort using an Illumina mated-pair library (Collins and Nyholm, 2011). Assemblies were annotated using the Rapid Annotation using Subsytem Technology (Aziz et al., 2008, RAST, rast.nmpdr.org) server. To search for Type IV secretion systems (T4SS), the VirB4 protein from *P. inhibens* DSM17395 was used to query the ANG isolate genomes using tblastn. Genomes were also analyzed with Anti-SMASH (Blin et al., 2013, Antibiotic and Secondary Metabolite Analysis Shell, antismash.secondarymetabolites.org) and BAGEL3 (van Heel et al., 2013, BActeriocin Genome mining tool, bagel.molgenrug.nl) for secondary metabolite and bacteriocin biosynthesis gene clusters. Draft genome assemblies have been deposited in DDBJ/EMBL/GenBank under accession numbers AFCF00000000 and JWLC00000000-JWLM00000000. The versions described in this manuscript are AFCF02000000 and JWLC01000000-JWLM01000000.

### TAXONOMIC ANALYSIS

A total of 79 genomes were used for analyses in this study. Fifty-seven *Roseobacter* genomes and 10 non-*Roseobacter* genomes were obtained from the NCBI ftp site (ftp://ftp.ncbi.nih.gov/genomes/, listed in Supplementary Figure 1). Twelve *Roseobacter* genomes are new to this study, including an improved assembly of the previously published *Leisingera* sp. ANG1 (**Table 1**). To ensure equal gene calling across the genomes, all genomes, including the 67 draft and completed genomes obtained from the NCBI ftp, were re-annotated using the RAST server (Aziz et al., 2008). Assembled contigs were reconstructed from the RAST-generated GenBank files for all genomes using the seqret application of the EMBOSS package (Rice et al., 2000).

**Table 1:** Genome assembly statistics for *Roseobacter* clade ANG isolates.

Isolate	Genome size (Mb)	# of genes	Missing genes* (% of total)	% GC	N50 (kb)	Contigs	Fold- coverage	Female ID
ANG-Vp	5.150	4,941	51 (1.0)	62.3	70	165	69.2	1
ANG-M1	5.375	5,097	63 (1.2)	62.0	211	180	132.3	3
ANG1	4.587	4,484	26 (0.6)	62.8	450	36	1,455^†^	1
ANG-DT	4.596	4,467	23 (0.5)	62.6	189	116	115.4	5
ANG-S	4.572	4,458	19 (0.4)	62.8	196	83	65.5	4
ANG-S3	4.597	4,468	18 (0.4)	62.7	300	84	129.0	2
ANG-M6	4.542	4,429	26 (0.6)	62.7	157	65	118.0	3
ANG-S5	4.660	4,534	33 (0.7)	62.5	233	54	123.5	2
ANG-M7	4.582	4,498	46 (1.0)	62.5	263	61	148.7	3
ANG-R	4.685	4,755	43 (0.9)	57.4	390	47	98.1	4
ANG-S4	4.538	4,619	9 (0.2)	57.2	978	20	71.9	2
ANG-S1	4.425	4,478	33 (0.7)	60.6	229	33	110.7	2

^*^As predicted by the RAST server (Aziz et al., 2008).

*^†^*Leisingera* sp. ANG1 was previously sequenced with an Illumina mate-pair library and is therefore and a much higher fold coverage than other genomes (Collins and Nyholm, 2011)*.

An initial survey of the *Roseobacter* clade was made using 51 ribosomal proteins. Queries were obtained from the BioCyc database (Caspi et al., 2010) for *Roseobacter denitrificans* OCh 114, excluding methyltransferases and putative proteins. Unlike many previous studies (Soucy et al., 2014) nucleotide sequences were used to potentially allow finer resolution of relationships. The top hits for each gene were aligned separately using MUSCLE (Edgar, 2004) and evaluated by hand to verify that the sequences were homologs. In-house python scripts created a concatenated alignment from all 51 genes. An optimal model of evolution was determined using the akaike information criterion with correction for small sample size (AICc). The program jModelTest 2.1.4. was used to compute likelihoods from the nucleotide alignment and to perform the AICc (Guindon et al., 2010; Darriba et al., 2012). The best-fitting model reported was GTR + Gamma estimation + Invariable site estimation. A maximum likelihood (ML) phylogeny was generated from the concatenated multi-sequence alignment using PhyML v3.0_360-500M (Guindon et al., 2010). PhyML parameters consisted of GTR model, estimated p-invar, 4 substitution rate categories, estimated gamma distribution, subtree pruning and regrafting enabled with 100 bootstrap replicates. This tree (Supplementary Figure 1) placed all of the new ANG isolates from this study into a single clade, corresponding to three groups (Clades 1, 2, and 4) previously described by Newton et al. (2010). Clade 4’s placement sister to clade 2 is discussed in Section “Results and Discussion.”

To further explore the relationships within these three clades a new scheme was devised. Forty-four genomes were selected from the clade, including all members corresponding to Newton’s Clade 1, for inclusion in this step. As most ribosomal proteins are quite short, only 18 ribosomal genes were used and 15 single-copy housekeeping genes were added. This offered the advantage of adding a net of ∼8,300 positions to the alignment, most of which are likely under less stringent selection than those of a ribosomal protein. An added advantage is that all 33 genes are shared with the Newton set. This creates a direct relationship facilitating comparison with that previous work. The top Blast hits for the 44 genomes were processed as described above for the ribosomal tree. The AICc test reported the same model for evolution as above. The tree was also generated using SPR and 100 bootstrap replicates. The resulting tree was rooted based on the ribosomal tree’s placement of the clades. This corresponded to the root being placed where Newton’s clades 1 and 2/4 diverge.

### AVERAGE NUCLEOTIDE IDENTITY

JSpecies1.2.1 (Richter and Rosselló-Móra, 2009) was used as described previously (Fullmer et al., 2014) to analyze the genomes for average nucleotide identity (ANI) and tetramer frequency patterns.

### SIDEROPHORE BIOCHEMICAL ASSAYS

To reduce contaminating iron, all glassware was washed and all solutions were prepared using water treated with a Nanopure Diamond filtration system (Barnstead, Lake Balboa, CA, USA). Siderophore production was confirmed using chrome azurol S (CAS) agar, modified for marine bacteria as previously described Whistler and Ruby (2003).

To test viability of ANG bacteria in iron-limiting conditions, several isolates were grown in the presence of the iron chelator ethylenediamine-N,N’-bis (2-hydroxyphenylacetic acid) (EDDHA) as described previously (McMillan et al., 2010). Cultures were grown for 24 h at 26°C in SWT then washed 3x in minimal sea salts solution (MSS, 50 mM MgSO_4_, 10 mM CaCl_2_, 350 mM NaCl, 10 mM KCl, 18.5 mM NH_4_Cl, 333 μM K_2_PO_4,_ FeCl_3_ 10 μM, 100 mM PIPES, pH = 7.2) with no added iron or EDDHA. Cultures were inoculated to an OD_600_ of 0.05 in MSS with 10 μM FeCl_3_. Glucose and casamino acids were added as carbon sources at 0.2 and 0.3% respectively and cultures were grown for 24 h at 26°C with shaking. To create iron-limiting conditions, EDDHA was added to the growth media at 10–30 μM. To test viability in iron-limiting conditions, cultures were grown for 24 h at 26°C, and the OD_600_ of each culture was measured and compared to control cultures without EDDHA. Siderophore production was measured from supernatants using the CAS liquid assay as described previously (Schwyn and Neilands, 1987). Further chemical characterization of siderophores was done using the Arnow (1937) and Csáky (1948) assays.

### HOMOSERINE LACTONE DETECTION

Homoserine lactone (HSL) production was detected using the HSL-sensing bacterium *Agrobacterium tumefaciens* NTL4 (pZLR4; Cha et al., 1998). To determine acyl homoserine lactone (AHL) production, we used a well-diffusion assay as previously described Ravn et al. (2001). Briefly, a 3-mL culture of *A. tumefaciens* NTL4 was grown for 24 h in LB with gentamicin 30 μg/mL at 28°C. One milliliter of this culture was used to inoculate 50 mL of AB minimal media containing 0.5% glucose and 0.5% casamino acids (Chilton et al., 1974). After a 24-h incubation, 100 mL of AB minimal media containing 1.2% agar was autoclaved. Once the molten agar had cooled sufficiently, glucose and casamino acids were added to 0.5% each and 5-bromo-4-chloro-3-indolyl-β-D-galactopyranoside (X-gal) was added to a final concentration of 75 μg/mL. The molten agar was then combined with the 24-h culture of *A. tumefaciens*, distributed into petri dishes and allowed to solidify.

To induce HSL production by ANG isolates, cultures were grown overnight at 26°C in either SWT or MSS with 30 μM FeCl_3_ and 0.5% of both glucose and casamino acids. To prevent the degradation of HSLs in alkaline conditions, the growth medium was buffered to pH 6.8 and never rose above 7.5 for any experiments. After a 24-h incubation, the cells were pelleted by centrifugation and the supernatant was filtered through a 0.22-μm filter. Wells were created in the *A. tumefaciens* agar plates using a sterile borer and 60 μL of cell-free supernatant was deposited into each well.

Accessory nidamental gland tissue was tested for the presence of AHLs by dissecting three separate ANGs from mature females as described above. Each ANG was homogenized in 300 μL of squid Ringer’s solution and the homogenate was centrifuged at 1,000 ×*g* for 10 min to pellet the ANG tissue. The supernatant containing bacterial cells was removed and centrifuged again at 10,000 ×*g* for 10 min and 60 μL of the resulting clarified homogenate was deposited in a well of the AHL detection plates. All AHL detection plates were incubated at 28°C and photographed after 48 h.

## RESULTS AND DISCUSSION

The genomes sequenced in this study were of a typical size for roseobacters, ranging from 4.4 to 5.4 Mb (**Table 1**). These large genomes are typical of the many cultured and sequenced organisms of the *Roseobacter* clade and reflect the diverse metabolisms reported in these bacteria (Newton et al., 2010). These data suggest that there has been little gene loss (or genome decay) as a result of close association with a host. However, several uncultivated roseobacters have streamlined genomes and may have a different lifestyle than most cultured members of this group (Luo et al., 2012, 2014). Many combinations of gene clusters for plasmid replication and partitioning were detected, particularly *repABC* genes. These data suggest that the ANG isolates have several extrachromosomal elements that may be resolved pending further sequencing efforts.

### TAXONOMIC ANALYSIS

**FIGURE 2:**
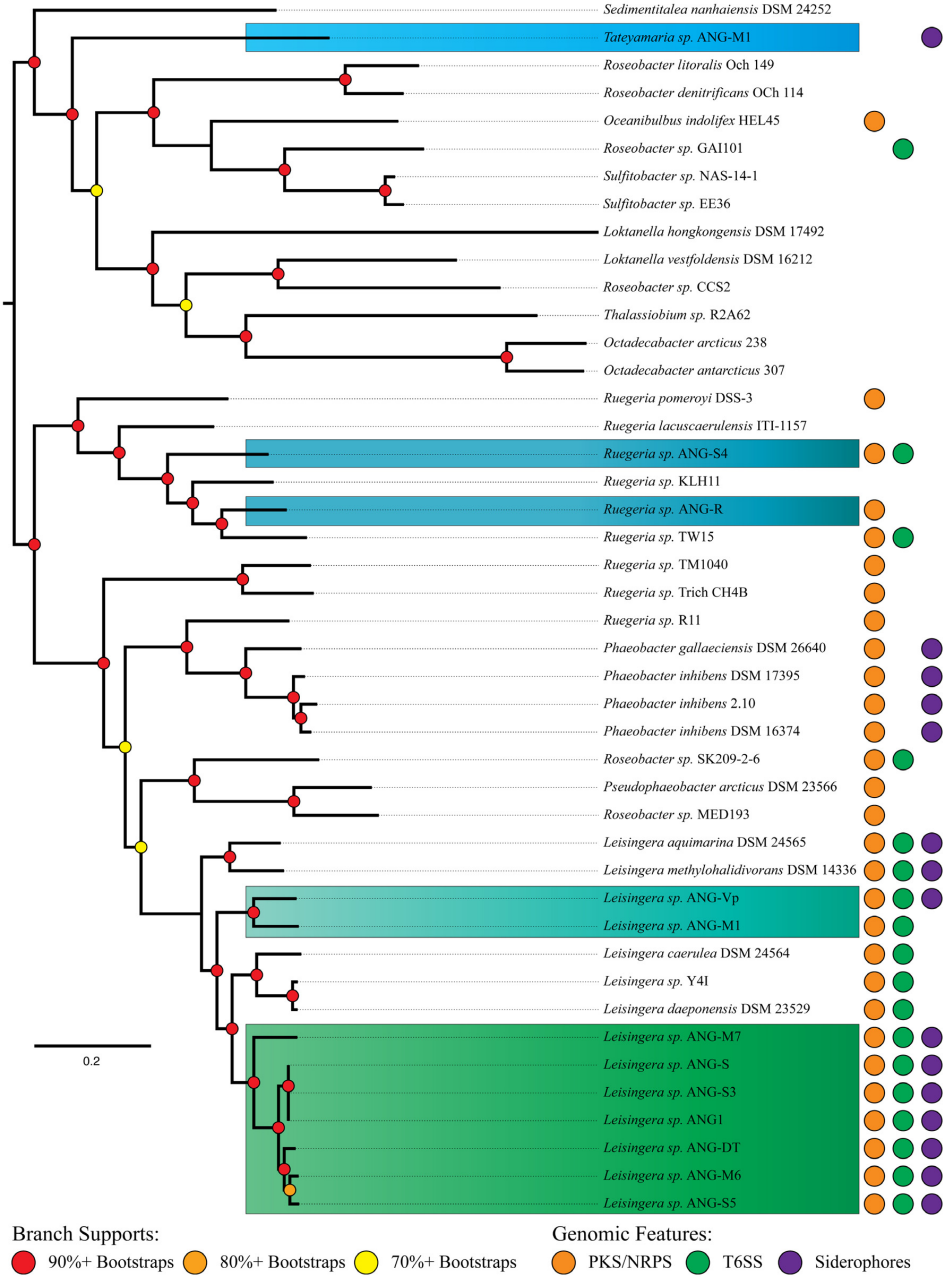
**MLSA analysis of *Roseobacter* clade isolates from the ANG with closely related organisms and distribution of significant gene clusters.** Phylogenetic analysis of 33 single-copy housekeeping genes places most ANG isolates in the previously described “Clade 1” of the *Roseobacter* clade (Newton et al., 2010). A polyketide/non-ribosomal peptide synthase gene cluster is distributed throughout most of the *Roseobacter* clade, while Type VI secretion systems and siderophores are limited to fewer members. Isolates are color-coded to indicate genus. Blue, *Ruegeria*; Light blue, *Tateyamaria*; Teal and green, *Leisingera*.

Of the ANG isolates identified, there were nine *Leisingera* (ANG1, ANG-DT, ANG-S, ANG-S3, ANG-S5, ANG-M6, ANG-M7, ANG-Vp, and ANG-M1), two *Ruegeria* (ANG-R and ANG-S4), and one *Tateyamaria* (ANG-S1) isolates. The 33 gene phylogenetic reconstruction placed these ANG isolates in five well-supported clades (**Figure 2**). The *Leisingera* isolates all grouped together in a single strongly supported clade, sister to four other described *Leisingera* taxa. This placement supports their recent designation as members of the *Leisingera* genus (Breider et al., 2014). The two *Ruegeria* ANG taxa did not place together, however, they are part of a clade composed of only *Ruegeria* taxa, affirming the putative genus designation. *Tateyamaria* placed on a basal branch long enough to suggest it is not closely associated with any of the taxa analyzed for this study.

The structure of the ribosomal tree (Supplmentary Figure 1) shares similarities with Newton et al.’s (2010) phylogeny. However, there are notable differences. First, the taxa of Newton’s clade 3 are split into two separate clusters. Second, all but one member of Newton’s clade 4 groups sister to clade 2. Finally, the two Rhodobacterales bacteria (HTCCs 2255 and 54623) fall among clades 2 and 4 rather than as part of the outgroups. The placement of clades 3 and 4 may be explained by the nature of gene concatenation. Concatenations can yield trees with high support values on topologies for which none of the constituents’ gene phylogenies match (Salichos and Rokas, 2013; Colston et al., 2014). Gene choice can result in significantly different well-supported topologies. Thus, the averaged history of the ribosome may have been “outvoted” by the average history of the balance of Newton’s seventy single-copy genes. The topology of the ribosomal tree was used to assign the root in the 33 gene tree (**Figure 2**) on the assumption that the ribosomal phylogeny was accurate in clade 4’s placement. The clade 4 taxa could be used as outgroups to clades 1 and 2 instead with no significant change to the further analyses of the ANG isolates.

The structure of the 33 gene tree (**Figure 2**) compares well with Newton’s phylogeny. Taxa previously identified as *Phaeobacter, Ruegeria, and Leisingera* formed polyphyletic clades. This occurrence was not unanticipated as the Newton et al. (2010) study showed a 70-gene tree with the same structure, albeit with fewer taxa. The genes analyzed in this study represent a subset of those analyzed in Newton et al. (2010) and therefore were expected to recapitulate this result. Our tree also aligns well with the recent reclassification by Breider et al. (2014). *Sedimentitalea nanhaiensis*, formerly *Leisingera nanhaiensis*, placed at the base of Newton’s clade 2, which is separated from the balance of the *Leisingera* genus. *Pseudophaeobacter arcticus*, formerly *Phaeobacter arcticus*, fell in a clade sister to the *Leisingera*, also isolated from the newly redefined *Phaeobacter* genus. Thus, its reclassification resolves a polyphyly observed in our tree. Likewise, *L. caerulea* and *L. daeponensis*, also reclassified from the *Phaeobacter* genus, resolve a separate polyphyly. As these two taxa are sister to established *Leisingera*, we find reassigning them to this genus in line with our results. The only remaining question of polyphyly in our 33 gene phylogeny is *Ruegeria* sp. R11, which groups with the *Phaeobacter/Pseudophaeobacter/Leisingera* clade. This isolate has been proposed as *Nautella* based on 16S rDNA similarity to the *Nautella* type strain and may not be a member of the *Ruegeria* genus (Fernandes et al., 2011).

**FIGURE 3:**
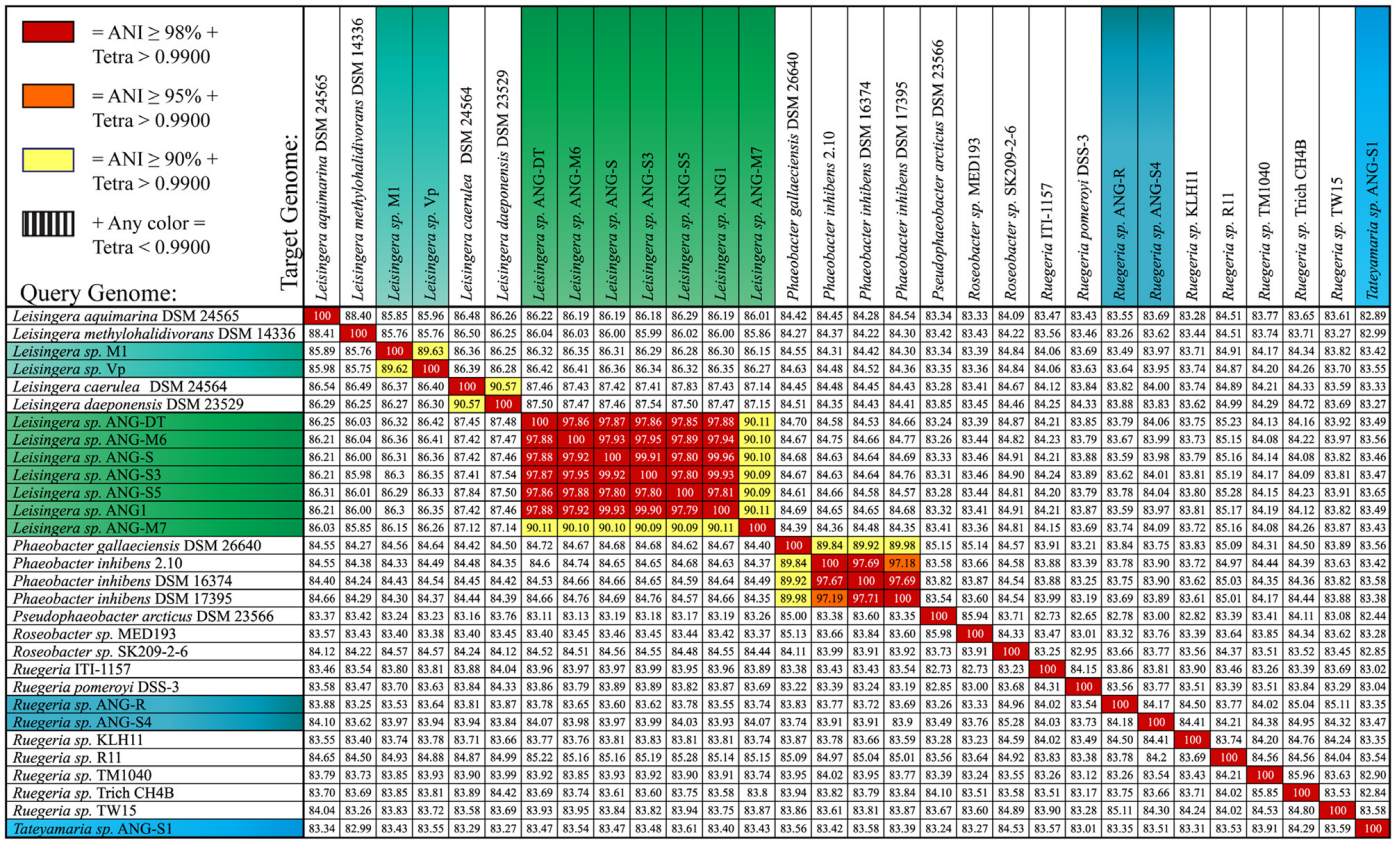
**Average nucleotide identity (ANI) comparison shows seven unique isolates from the ANG.** Six of the isolates cultured from the ANG are highly similar, sharing >98% ANI (ANG-DT, ANG-M6, ANG-S, ANG-S3, ANG-S5, and ANG1). These isolates dominate the culturable isolates of the ANG and have been consistently isolated from five different animals. These isolates appear to be a novel species while isolate ANG-M7 may be a second novel *Leisingera* taxon. Isolates ANG-Vp and ANG-M1 share 90% ANI but each appear to represent a novel taxon. The other isolates (ANG-R, ANG-S4, and ANG-S1) are unique from other sequenced roseobacters. Isolates are color-coded to indicate genus. Blue, *Ruegeria*; Light blue, *Tateyamaria*; Teal and green, *Leisingera*.

The phylogenetic analyses identified apparent relationships at approximately the genus level. In order to attempt to refine these results and provide species-level putative designations, ANI was employed using the accepted ANI cutoff of 95% (**Figure 3**, Konstantinidis et al., 2006; Richter and Rosselló-Móra, 2009). The ANG isolates fall into seven putative species groups. Six *Leisingera* isolates (ANG1, ANG-DT, ANG-S, ANG-S3, ANG-S5, and ANG-M6) formed one group, and three other *Leisingera* isolates (ANG-M7, ANG-M1, ANG-Vp) and the two *Ruegeria* isolates each formed its own singleton group. The *Leisingera* isolates are of particular interest as previous research has shown that the most common symbionts within the ANG belong to the genus *Leisingera,* though they were previously classified within the genus *Phaeobacter* (Collins and Nyholm, 2011; Collins et al., 2012). One putative species of *Leisingera* was consistently isolated from the five individual ANGs used in this study. This cluster of isolates likely represents the dominant culturable symbiont present in the ANG and includes the previously sequenced isolate, *Leisingera* sp. ANG1. Notably, the ANI values of the ANG isolates all fell short of even 90% identity with any of the previously described species. These data suggest that each of these putative ANG species is, indeed, a novel taxon. Providing comprehensive polyphasic species descriptions is beyond the scope of this work, so we propose these taxa as sp. of their various assigned genera.

### RECLASSIFICATION OF *Phaeobacter gallaeciensis* ANG1

Consistent with previous research, our results suggest the isolate we had previously identified as *P. gallaeciensis* is phylogenetically distinct from the type species, *P. gallaeciensis* DSM 26640 (Thole et al., 2012; Breider et al., 2014). We therefore reclassify the isolate *P. gallaeciensis* ANG1 as *Leisingera* sp. ANG1 pending further phenotypic analyses.

### GENOME CHARACTERISTICS AND GENERAL METABOLISM

Of the 12 ANG symbionts examined in this study, all have genes encoding a complete Entner–Doudoroff pathway for metabolizing glucose. Furthermore, all of them lack the gene for phosphofructokinase, a key enzyme from the Embden–Meyerhof–Parnas pathway. This is typical of many previously sequenced and complete genomes from the *Roseobacter* lineage (Moran et al., 2004; Newton et al., 2010; Wagner-Döbler et al., 2010). Two organisms (*Tateyamaria* sp. ANG-S1 and *Ruegeria* sp. ANG-S4) contain all genes for a complete pentose-phosphate pathway. The others contain most genes for the pathway, with the exception of a gene encoding 6-phosphogluconate dehydrogenase. As an alternative metabolic pathway, 6-phosphogluconate produced by the first two enzymes of the pentose phosphate pathway could feed into the Entner–Doudoroff pathway for further carbohydrate metabolism (Fuchs, 1999; Berger et al., 2014).

While the *Roseobacter* clade was first described as a group of obligate aerobic organisms, recently it has been shown that some members contain enzymes needed for anaerobic respiration of nitrate (Dogs et al., 2013). All of the isolates from the ANG contain the gene for nitrate reductase that could be used for anaerobic respiration of nitrogen. Most isolates, with the exception of *Tateyamaria* sp. ANG-S1, also contain genes for other denitrifying enzymes to further reduce nitrogenous oxyanions. These data suggest that the ANG isolates may be able to survive and thrive in anaerobic environments by respiring nitrogenous oxyanions.

Although genes associated with phototrophy were detected in *Tateyamaria* ANG-S1, including bacteriochlorophyll a, these genes were not detected in the other ANG isolates. These data are consistent with previous observations of Clade-1 roseobacters which were not found to be phototrophic (Newton et al., 2010; Luo and Moran, 2014).

### PROTEIN SECRETION SYSTEMS

While a Type IV secretion system is present in many roseobacters, we detected *virB* in only two of the genomes examined here (ANG-M1 and ANG-R). Previous literature has suggested these systems are used for communication between bacteria and eukaryotic cells (Luo and Moran, 2014). However, given that a large proportion of isolates from the ANG appear to lack this system, the T4SS may not be a critical means of communication between the consortium and its host.

An interesting feature of the *Leisingera* genus is that all sequenced genomes contain genes for a Type VI secretion system (T6SS, **Figure 2**). In *L. daeponensis* and *L. caerulea* it has been shown that this T6SS exists on a plasmid (Beyersmann et al., 2013; Dogs et al., 2013). In *Leisingera* sp. ANG1 the T6SS is located on a large contig (>500 kb) containing *repAB* plasmid partitioning genes, suggesting that the T6SS in this species is also located on a plasmid. Similar conclusions were reached with the genomes of *L. caerulea, L. daeponensis, L. methylohalidivorans,* and *L. aquimarina*. Each of these organisms has genes for a T6SS on plasmids that vary in size (from 109 kB in *L. caerulea* to 526 kB in *Leisingera* sp. ANG1); however, all have a DnaA 1-like replicase (Beyersmann et al., 2013; Buddruhs et al., 2013; Dogs et al., 2013; Riedel et al., 2013). While other roseobacters contain a T6SS, the conservation of the T6SS on similar plasmids could be characteristic of this genus.

Several functions of the T6SS have been proposed, including antimicrobial roles, as evidenced by direct cell-contact mediated killing (Murdoch et al., 2011; Russell et al., 2011). The T6SS has also been shown to be involved with host–microbe interactions, particularly in the *Rhizobiales*. *A. tumefaciens* shows attenuated ability to create crown gall tumors when the T6SS is deleted (Wu et al., 2008). Similarly, the nitrogen-fixing plant symbiont *Rhizobium leguminosarum* lacking a T6SS will successfully colonize its host, however, it will fail to fix nitrogen (Bladergroen et al., 2003). The T6SS has also been implied in many other general associations between microorganisms, including predator evasion (Pukatzki et al., 2006) and self/non-self recognition (Gibbs et al., 2008).

It is interesting that all of the isolates, with one exception (*Ruegeria* sp. ANG-R), have genes for a T6SS, including isolates outside of the *Leisingera* genus. This suggests that the T6SS in these bacteria may be important for communication with the host and/or with other bacteria. In the ANG of *E. scolopes,* bacteria are housed in high densities within the epithelium-lined tubules of the organ (Collins et al., 2012). Such high densities of bacterial cells foster close contact with other bacteria and many host cells, including the ANG epithelium and hemocytes, the principle cellular innate immunity component of the host. Given that the T6SS functions by direct cell-to-cell contact, it would be an ideal mechanism for the delivery of effectors directly to other symbionts and/or host tissues. The T6SS may play a role in mediating how these organisms are selected from the environment and explain how some species are able to dominate the bacterial populations within a given tubule (Collins et al., 2012).

### SECONDARY METABOLITES

Members of the *Roseobacter* clade have been shown to produce several unique secondary metabolites. Some of the most notable ones include antibacterials such as TDA, produced by organisms such as *P. inhibens* and *Ruegeria* sp. TM1040, and the blue pigment indigoidine, produced by organisms such as *Leisingera* sp. Y4I and *L. daeponensis* (Geng et al., 2008; Cude et al., 2012). None of the biosynthetic genes for either of these compounds were found in any of the genomes sequenced. Furthermore, no classical antibiotic synthesis pathways (e.g., tetracycline, carbapenems, etc.) were found.

However, analysis with the Antibiotic and Secondary Metabolite Analysis Shell (AntiSMASH, Blin et al., 2013) revealed several gene clusters encoding potential secondary metabolism (**Table 2**). These included gene clusters for siderophore synthesis, autoinducer synthases (*luxI* homologs), polyketide/non-ribosomal peptide synthases (PKS/NRPS) and production of volatile compounds such as terpenes. The BActeriocin GEnome mining tooL (BAGEL, van Heel et al., 2013), was used to screen genomes for possible bacteriocin producing gene clusters, which were found in the *Ruegeria* isolates (ANG-R and ANG-S4) as well as *Tateyamaria* sp. ANG-S1 (**Table 2**). Bacteriocins are a broad group of proteins that can be used to kill other bacteria but have also been shown to act as inducers of invertebrate metamorphosis and thus may serve a number of functions (Cotter et al., 2013; Shikuma et al., 2014).

**Table 2:** Secondary metabolite gene clusters detected with AntiSMASH and BAGEL.

	PKS/NRPS	LuxRI	Bacteriocin	Siderophore	Terpene	Ectoine
*Leisingera* sp. ANG-Vp	1	1	0	1	0	1
*Leisingera* sp. ANG-M1	1	1	0	0	0	1
*Leisingera* sp. ANG1	1	1	0	1	0	0
*Leisingera* sp. ANG-DT	1	1	0	1	0	0
*Leisingera* sp. ANG-S	1	1	0	1	0	0
*Leisingera* sp. ANG-S3	1	1	0	1	0	0
*Leisingera* sp. ANG-M6	1	1	0	1	0	0
*Leisingera* sp. ANG-S5	1	1	0	1	0	0
*Leisingera* sp. ANG-M7	1	1	0	1	0	0
*Ruegeria* sp. ANG-R	1	2	3	0	0	1
*Ruegeria* sp. ANG-S4	2	2	3	0	0	0
*Tateyamaria* sp. ANG-S1	0	1	2	1	1	0

All isolates have a conserved non-ribosomal peptide/polyketide synthase gene cluster characterized previously (**Table 2**, Martens et al., 2006). This gene cluster is conserved in the *Roseobacter* lineage, being found in 28 of 57 previously sequenced genomes, and is comprised of four genes: a non-ribosomal polypeptide synthase, a polyketide synthase, a glycosyltransferase and a phosphopantetheinyl transferase. However, the product of this gene cluster has not yet been characterized. Given that this gene cluster is well-conserved throughout the *Roseobacter* lineage, its product and function should be elucidated through future experiments.

### QUORUM SENSING

Homoserine lactones produced by LuxI homologs have been widely studied as quorum sensing molecules in bacteria, including the *luxIR* system of *V. fischeri*, the light organ symbiont of *E. scolopes* (Antunes et al., 2007; Miyashiro and Ruby, 2012). AntiSMASH detected 2 separate pairs of *luxIR* homologs in the ANG isolates that were most similar to the *ssaIR* and *ssbIR* previously described in *Ruegeria* sp. KLH11 (Zan et al., 2012). However, only the *Ruegeria* isolates, ANG-S4 and ANG-R, have both pairs of *luxIR* homologs. Most of the ANG bacteria only have homologs of *ssbIR*. In *Ruegeria* sp. KLH11, these two systems work together to control biofilm formation and motility (Zan et al., 2012). The genes *ssaI* and *ssaR*, are shown to regulate the change between adherent and planktonic lifestyles. Increased levels of HSLs promote flagellar growth and motility, while lower levels foster biofilm development. The actions of these genes can be indirectly repressed by *ssbIR*. The fact that so many ANG isolates have only the *ssbIR* homologs suggest that there may be a unique function for these quorum sensing genes independent of the *ssaIR* quorum sensing system. In addition to *ssbIR*, the ubiquitous *luxIR* homologs in the *Roseobacter* genomes from the ANG are also similar to the *raiIR* genes described in *Rhizobium etli* (Rosemeyer et al., 1998). Both SsbIR and RaiIR are known to produce 3-hydroxyl-HSL compounds, but *raiIR* has been shown to control growth and nitrogen fixation, not motility. This raises the possibility that the *luxIR* genes in ANG roseobacters may regulate growth of bacteria within the ANG.

**FIGURE 4:**
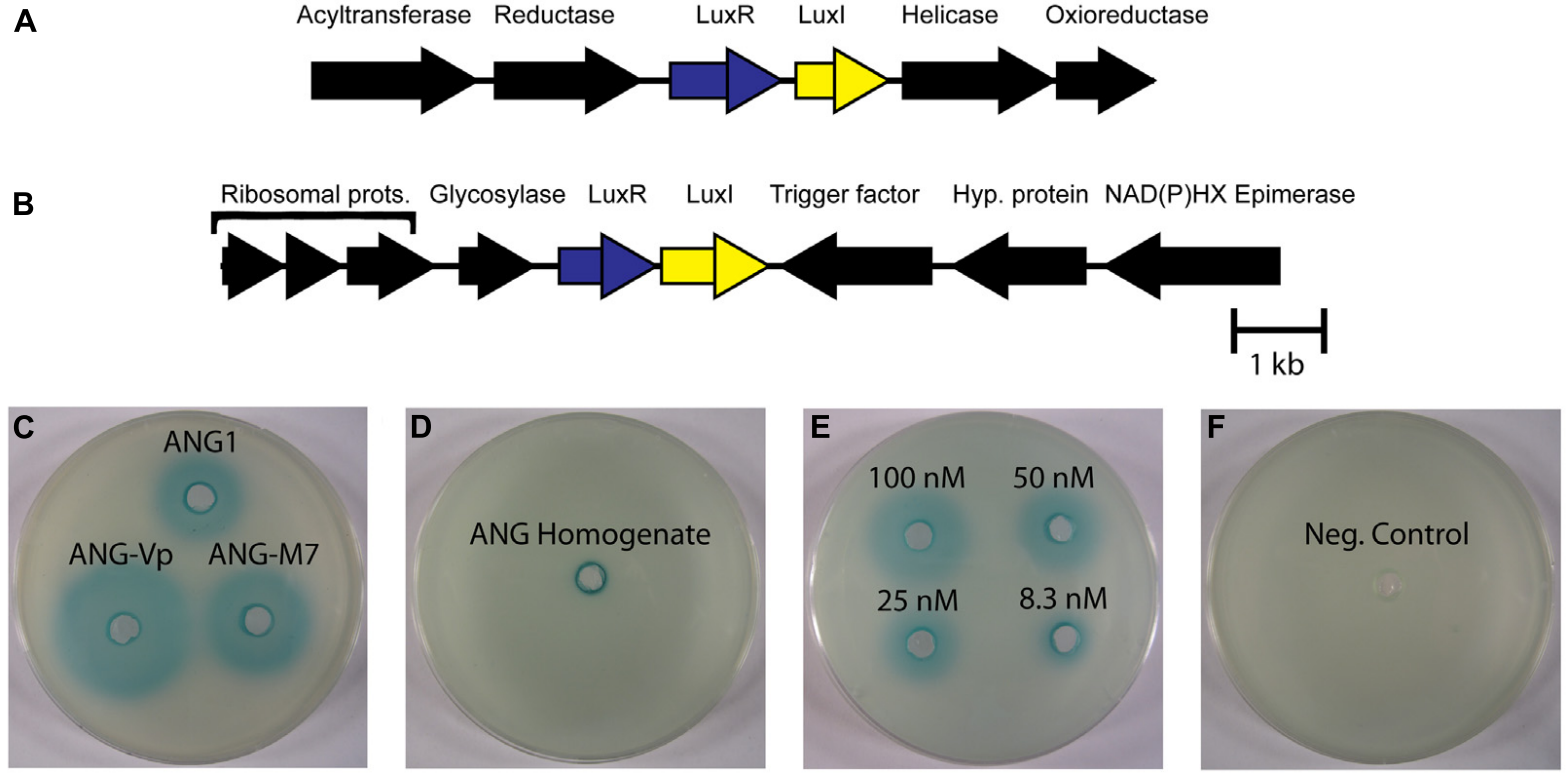
**LuxIR homologs in *Roseobacter* clade organisms from the ANG and associated homoserine lactone production. (A)** All ANG isolates have a pair of *luxIR* homologs flanked by potential anabolic genes (crontonyl CoA reductase and acetyltransferase) and a helicase and oxioreductase. **(B)** Another pair of *luxIR* homologs is present only in the *Ruegeria* species (isolates ANG-S4 and ANG-R) flanked by ribosomal proteins, a cell division trigger factor and a glycosylase. **(C)** Representative picture of β-galactosidase activity induced by homoserine lactone in supernatants from cultures of *Leisingera* sp. ANG1, *Leisingera* sp. ANG-M7, and *Leisingera* sp. ANG-Vp. **(D)** Homoserine lactones were also detected in ANG homogenates (representative image, *n* = 3 separate ANGs). **(E)** Semi-quantitative dilution of N-3-oxo-hexanoyl-homoserine lactone. **(F)** 60 μL growth medium (Negative control).

To determine if HSLs are present in the ANG and are produced by the bacterial symbionts, we tested for the presence of AHLs using a semi-quantitative biosensor assay. All isolates that could grow to high density in liquid medium produced detectable HSLs (**Figure 4**). Species like *Tateyamaria* sp. S1 did not grow to a very high density and failed to produce enough HSL to be detected by the assay (not shown). The homogenates of three ANGs were also tested and resulted in small zones of β-galactosidase activity around the assay wells, suggesting that HSLs are produced in the ANG and could contribute to the symbiosis by influencing gene expression of the bacterial consortium. As a negative control, host gill tissue was also homogenized in a similar manner to ensure that compounds from squid tissue were not inducing expression of β-galactosidase in the *A. tumefaciens* biosensor. No enzymatic activity was observed in this control (not shown), confirming the specificity of the assay.

While HSLs were detected in both pure culture and in ANG homogenate, gene regulation by HSL quorum sensing may be different than what has been described for their nearest homologs in *Ruegeria* sp. KLH11. Most ANG isolates, including the dominant *Leisingera* species, lack the *ssaIR* homologs directly responsible for the increase of motility described in *Ruegeria* sp. KLH11. This suggests there is a yet undescribed role for the *ssbIR* homologs in the *Roseobacter* clade isolates from the ANG.

Future research should investigate the chemical nature of the HSL produced by the autoinducer synthases in individual ANG isolates. The nearest characterized homologs, both RaiI in *R. etli* and SsbI in *Ruegeria* sp. KLH11 produce 3-hydroxyl-HSL compounds (Rosemeyer et al., 1998; Zan et al., 2012). Future studies should confirm that the members of the ANG consortium also produce this type of HSL. Genetics have not yet been developed in any of the cultured ANG isolates, however, creating a non-functioning mutant of the autoinducer synthase could reveal phenotypes controlled by quorum sensing. Comparing transcriptomes between HSL^-^ and wild type strains may also reveal genes that are controlled by quorum sensing. Moreover, previous research has shown that these symbionts are likely environmentally transmitted (Kaufman et al., 1998). Thus, the symbionts encounter three environments of varying cell density, from ambient seawater with a low density of symbionts, to the tubules of the ANG where the cells are highly concentrated, to the egg jelly coat with a lower density. Given the profound differences in cell density between free-living symbionts in seawater and the tubules of an ANG, quorum sensing may be an ideal mechanism for gene regulation between the different environments experienced by ANG bacteria (host/ANG, egg, free-living). Further studies should also examine how gene expression changes from the high-cell density environment of the ANG to the egg jelly coat, where cell densities will be lower, but where any anti-fouling compounds may be produced.

### SIDEROPHORES

Another group of secondary metabolite biosynthesis genes that was detected in the genomes of ANG isolates were siderophores. Siderophores are small molecules with high affinities for iron and can be used by bacteria for iron scavenging. Iron is needed for many cellular functions, including respiration, detoxification of reactive oxygen species (e.g., catalases, super-oxidase dismutase), and metabolism (e.g., aconitase of the TCA cycle). Very few organisms are known to survive without iron (Andrews et al., 2003). One way that bacteria can acquire iron in environments where it is a limiting resource is by producing siderophores to sequester iron from other sources.

**FIGURE 5:**
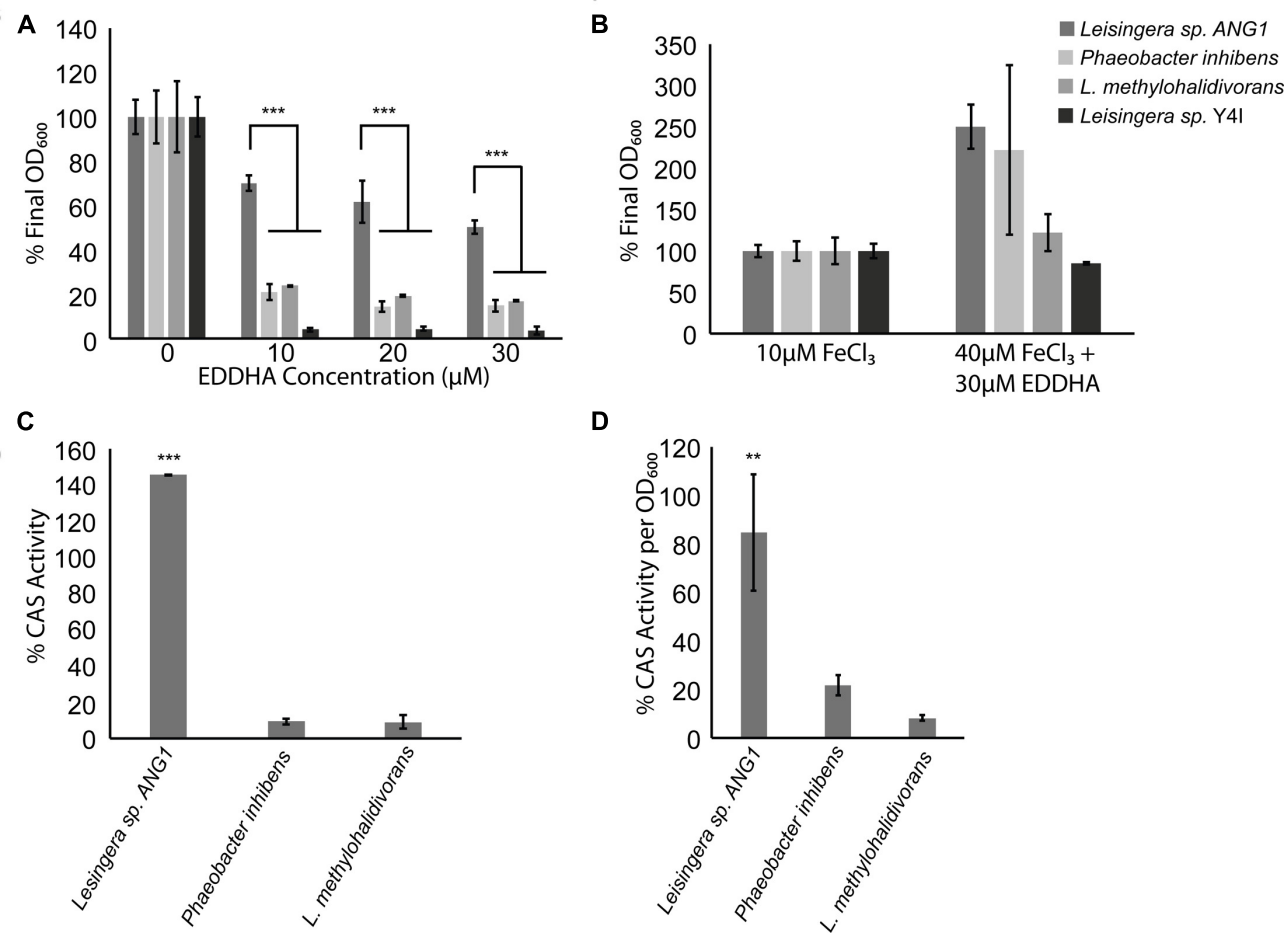
***Roseobacter* clade symbionts from the ANG have a growth advantage in iron-limiting conditions, possibly due to siderophore production. (A)** While other *Roseobacter* clade organisms were inhibited by the presence of an iron-chelator, *Leisingera* sp. ANG1 grew to more than 50% of its optical density even if EDDHA was at three times the concentration of available iron. **(B)** EDDHA is not toxic to *Roseobacter* clade organisms, as adding enough iron to overwhelm the chelator restored the growth defect to all *Roseobacter* clade organisms. **(C)** *Leisingera* sp. ANG1 produced more siderophore than non-ANG isolates in the presence of EDDHA. **(D)** Even if no iron chelator were present, siderophores were more abundant in the supernatants from cultures of *Leisingera* sp. ANG1. ** *p* < 0.01, *** *p* < 0.001.

Siderophore synthesis genes in the *Roseobacter* clade are rare. Of previously sequenced *Roseobacter* genomes, only six genomes from four species (*L. aquimarina*, *L. methylohalidivorans*, *P. inhibens,* and *P. gallaeciensis*) are predicted to have siderophore synthesis genes (**Figure 2**). However, all roseobacters isolated from the ANG of *E. scolopes*, with the exception of *Ruegeria* sp. ANG-R and ANG-S4, have either siderophore biosynthesis genes or showed siderophore activity in biochemical assays (**Table 2** and **Figure 5**). For example, *Leisingera* sp. ANG-M1 had no predicted siderophore synthesis genes, but siderophore activity was detected when grown on CAS agar and in CAS liquid assays, suggesting that these biosynthetic genes may not be annotated, perhaps due to the fragmented state of the assembled genome for this isolate. Conversely, *Tateyamaria* sp. ANG-S1 has siderophore biosynthetic genes, but failed to show siderophore activity (not shown). Taken together, these data suggest induction of siderophore synthesis genes may be controlled very differently in *Tateyamaria* sp. ANG-S1 and may be induced only under specific conditions.

We compared growth and siderophore production in iron-limiting conditions of *Leisingera* sp. ANG1, a representative of the dominant ANG symbionts, to three other species from the *Roseobacter* lineage. Siderophore-producing strains *P. inhibens* DSMZ 17395 and *L. methylohalidivorans* DSM 14336 were tested along with the non-siderophore producing strain *Leisingera* sp. Y4I. When grown in the presence of the iron chelator EDDHA, most roseobacters had a growth defect, growing to only 20% of the control density (**Figure 5A**). However, *Leisingera* sp. ANG1 had a much smaller growth defect (*p* < 0.001), growing to greater than 50% of the control OD when concentrations of EDDHA were three times the concentration of available iron in the media (**Figure 5A**). To show this was not due to a toxic effect of EDDHA, FeCl_3_ was added to higher concentrations (40 μM) to overwhelm the iron chelator, which restored the growth of all organisms (**Figure 5B**).

The survival of *Leisingera* sp. ANG1 under iron-limiting conditions could be due to the higher levels of siderophores produced by these organisms. Supernatants from cultures of strains that failed to grow (*P. inhibens* and *L. methylohalidivorans*) showed very little CAS activity while supernatants from cultures of ANG1 had very high levels of CAS activity, indicative of a high concentration of siderophores (*p* < 0.001, **Figure 5C**). To determine if this increase was a consequence of the increased growth of *Leisingera* ANG1, CAS activity was measured in supernatants from cultures without any iron chelator added. This allowed the bacteria to grow and deplete the iron available in the media, leading to induction of siderophore synthesis. Supernatants from cultures of *Leisingera* sp. ANG1 had more CAS activity than either *P. inhibens* DSM17395 or *L. methylohalidivorans* DSM14336 per unit OD_600_ (*p* < 0.01, **Figure 5D**). These data suggest that the abundance of siderophores produced by *Leisingera* sp. ANG1 is not just due to an increase in cell number, but instead to increased siderophore production at the cellular level.

**FIGURE 6:**
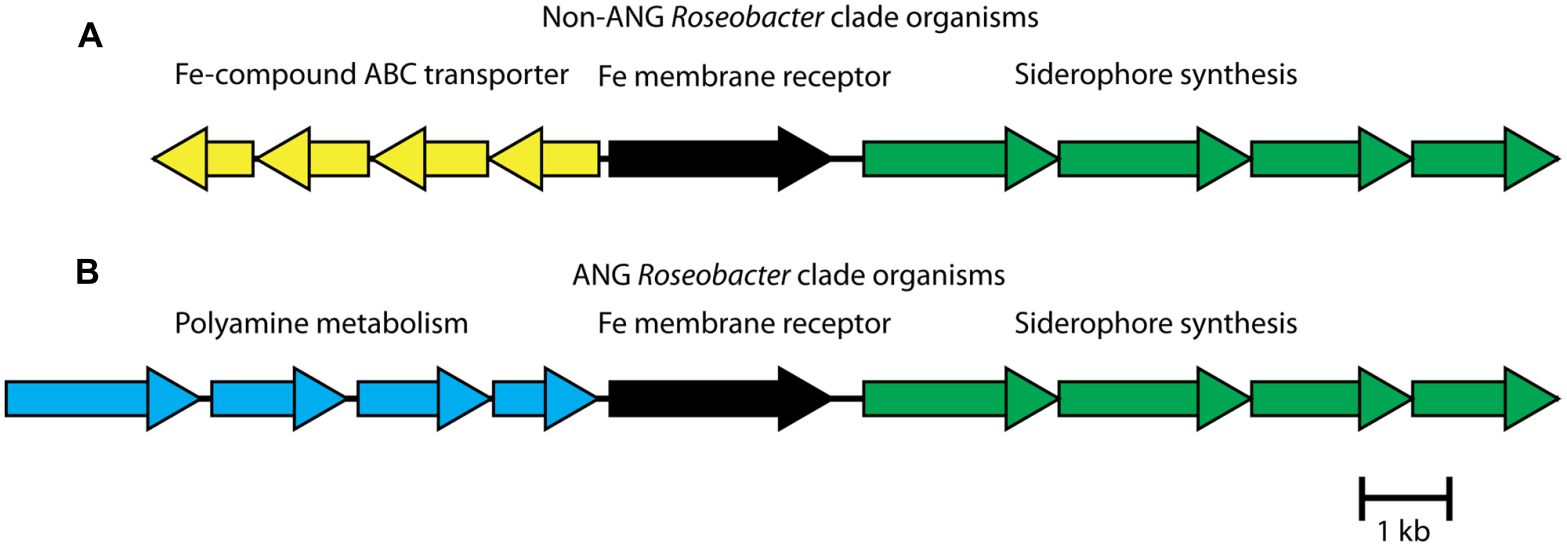
***Roseobacter* isolates from the ANG have a unique genome rearrangement upstream of siderophore biosynthesis group. (A)** In previously sequenced *Leisingera* and *Phaeobacter* species, an ABC transporter, predicted to transport iron compounds like iron dictate, lies upstream of a membrane receptor protein and the siderophore synthesis gene cluster. **(B)** In ANG bacteria, polyamine metabolism genes replace the ABC transporter genes. In addition to modifying regulatory elements, the four polyamine genes could synthesize a polyamine backbone of the siderophores, such as putrescine.

Examining the siderophore biosynthesis genes in roseobacters isolated from the ANG, revealed a unique genome rearrangement (**Figure 6**). In all other siderophore-producing roseobacters, siderophore synthesis genes are located downstream of an iron membrane receptor and an iron-compound ABC transporter. In roseobacters isolated from the ANG, four genes related to polyamine metabolism are inserted upstream of the iron membrane receptor (**Figure 6**). The polyamine genes upstream of the siderophore synthesis cluster are sufficient to synthesize putrescine, a backbone of certain catechol siderophores such as photobactin from *Photorhabdus luminescens* (Ciche et al., 2003). Testing the supernatant of *Leisingera* sp. ANG1 with the Arnow assay showed that catechol siderophores were being produced. This genome rearrangement may be responsible for the higher production of siderophores in *Leisingera* sp. ANG1, perhaps by altering the regulatory elements upstream of the siderophore biosynthesis genes or perhaps by coupling the production of putrescine and the catecholate siderophore. Future research may determine if putrescine is a structural component of the catechol siderophores produced by the ANG symbionts such as the dominant *Leisingera* symbionts.

Producing siderophores can be beneficial to bacteria that colonize animal tissues. Iron-chelating proteins produced by hosts can effectively deplete freely available iron to the associated microbiota (Ong et al., 2006). Furthermore, a host infected with a pathogen will sometimes increase production of iron-chelating proteins as a way to starve infectious bacteria of a critical resource (Jurado, 1997). One of the most-widely studied models is the siderophore enterobactin which is produced by several species of enteric bacteria, including *Salmonella* and *Escherichia* species (Raymond et al., 2003). This iron-chelating molecule acquires iron from serum proteins carrying iron, such as transferrin, and the siderophore-iron complex is taken up by the infecting bacteria to keep them supplied with iron. To combat this, the innate immune system produces proteins to bind siderophores in order to prevent the iron-scavenging molecules from fulfilling their purpose (Goetz et al., 2002; Abergel et al., 2008).

In invertebrates, iron sequestration can be performed by two ubiquitous proteins, ferritin, and transferrin. Ferritin is present in the hemolymph of invertebrates where it can function as an iron transporter or iron scavenger (Ong et al., 2005) and transferrin is up-regulated in insect epithelia during bacterial infection (Buchon et al., 2009; Wang et al., 2009). Both of these proteins have been found in transcriptomic and proteomic data from both hemocytes and light organ tissues of *E. scolopes* (Schleicher and Nyholm, 2011, Collins, unpublished data). These iron chelators, if present in the ANG, could provide a selective pressure that other roseobacters would have to overcome. In such a case, siderophore-producing organisms such as *Leisingera* sp. ANG1 may have an advantage over other bacteria and this may contribute to its dominance in the consortium. Colonization of cephalopod ANGs is likely via environmental transmission (Kaufman et al., 1998) and overcoming iron-limitation may be one part of what is likely a complex process for establishment and development of the association.

The function of the ANG and its bacterial consortium remains unknown even though it was hypothesized that the bacteria deposited in the jelly coats of squid eggs may play a role in protecting the egg masses from fouling, possibly through the production of antimicrobial compound(s) (Biggs and Epel, 1991). Previous research in the eggs of the shrimp (*Palaemon macrodactylus*) have shown that, once the eggs are brooded, *Alteromonas* sp. bacteria colonize the surface of the egg and produce the antimicrobial compound 2, 3-indolinedione that protects the eggs from fungal infection (Gil-Turnes et al., 1989). However, shrimp eggs acquire these epibionts from seawater which is an important distinction from squid eggs, where the bacterial symbionts from the ANG are actively deposited into jelly coat layers. Future research will attempt to understand the role of these bacteria within the eggs of developing embryos and try to discern what contribution they may make to deter fouling organisms.

This study sets the foundation for future research on the ANG symbionts by characterizing the genomes of several isolates from the *Roseobacter* lineage. We have identified many features of these genomes that may be important in the ANG association including Type VI secretion systems, siderophore production and putative quorum sensing systems using HSLs. The ANG and associated roseobacters are found worldwide in many different cephalopod species. This trend suggests that the consortium may play a similar and conserved role in squid and cuttlefish. Future research will hopefully elucidate the contribution of these bacteria to the development and survival of cephalopods and their embryos. Genome analyses of the *Roseobacter* clade bacteria that dominate the ANG, along with future genomic and transcriptomic studies of other ANG symbionts and the entire consortium will provide a number of exciting avenues of research to help elucidate the nature of this widely distributed association.

## Conflict of Interest Statement

The authors declare that the research was conducted in the absence of any commercial or financial relationships that could be construed as a potential conflict of interest.
