# Supplementary material for: Comparative genomics of Roseobacter clade bacteria isolated from the accessory nidamental gland of Euprymna scolopes
Source: Front Microbiol. 2015 Feb 23;6:123. doi: 10.3389/fmicb.2015.00123 (PMC4337385; doi:10.3389/fmicb.2015.00123)
Supplement: Supplementary file 1 [file image_1_(1).pdf]

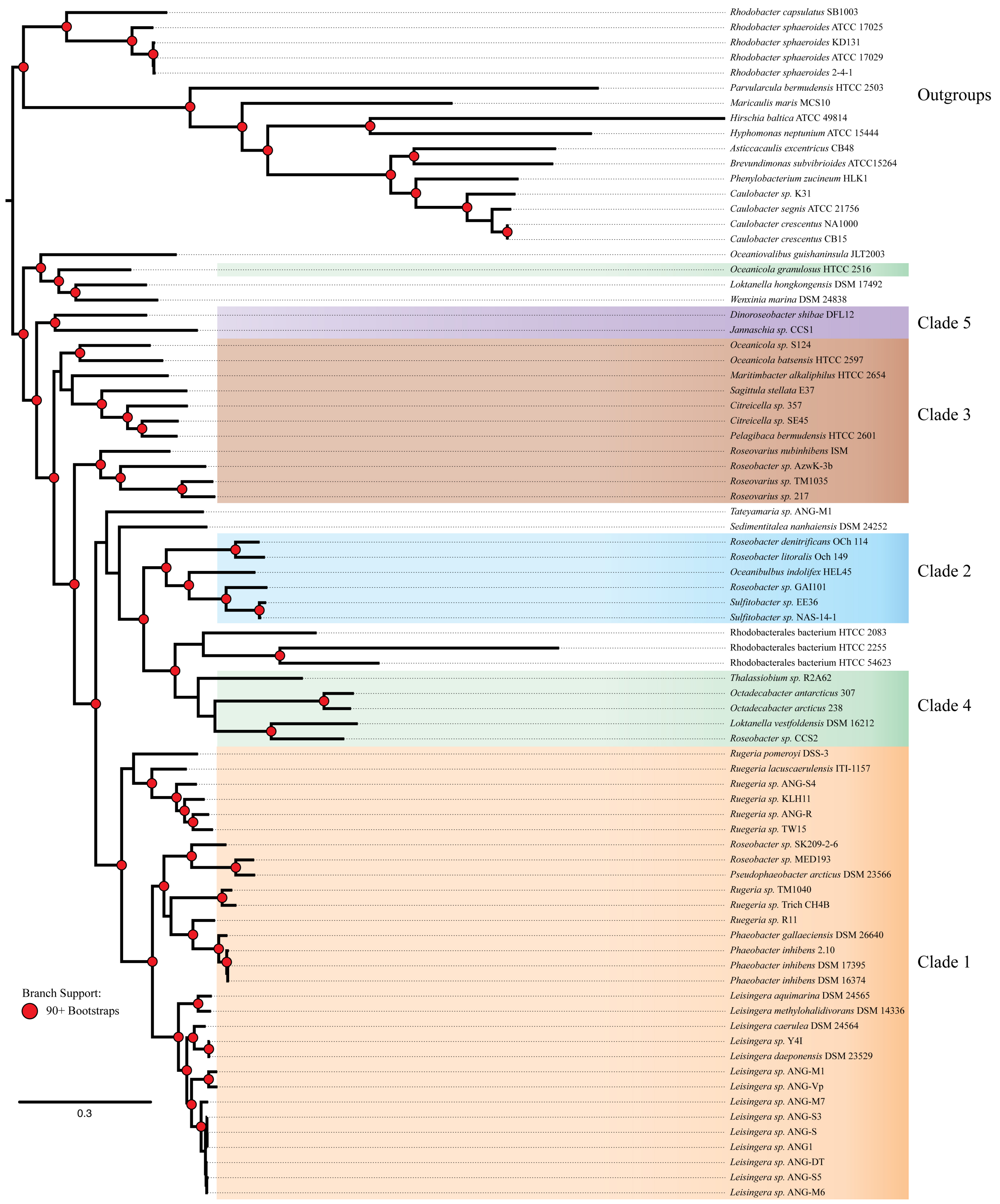

Supplementary Figure 1. Ribosomal protein phylogeny of the *Rosebacter* clade. Phylogenetic analysis of 51 ribosomal proteins results in a topology with the ANG isolates grouping in two large two sister clades. Organisms are colored by which of the five clades they fell into as reported by Newton et. al (Newton et. al., 2010). The three "Rhodobacterales bacteria" appear to be long-branch artifacts in this phylogeny and when included in the thirty-three gene topology (Figure 2). HTCC 2083 exists on a very long branch with lesser support than many of the other branches in Newton's phylogeny. Thus, all three were removed from the dataset in further analyses.
